# Autologous cytokine-induced killer cell transfusion increases overall survival in advanced pancreatic cancer

**DOI:** 10.1186/s13045-016-0237-6

**Published:** 2016-02-03

**Authors:** Zibing Wang, Yuqing Liu, Rui’e Li, Yiman Shang, Yong Zhang, Lingdi Zhao, Wei Li, Yonghao Yang, Xiaojie Zhang, Tiejun Yang, Changfu Nie, Feng Han, Ying Liu, Suxia Luo, Quanli Gao, Yongping Song

**Affiliations:** Department of Immunotherapy, Affiliated Cancer Hospital of Zhengzhou University and Henan Cancer Hospital, Zhengzhou, 450008 China; Department of Oncology, Third Affiliated Hospital of Xinxiang Medical College, Xinxiang, 453003 China; Department of Urology, Affiliated Cancer Hospital of Zhengzhou University and Henan Cancer Hospital, Zhengzhou, 450008 China; Department of Hepatobiliary and Pancreatic Surgery, Affiliated Cancer Hospital of Zhengzhou University and Henan Cancer Hospital, Zhengzhou, 450008 China; Department of Oncology, Affiliated Cancer Hospital of Zhengzhou University and Henan Cancer Hospital, Zhengzhou, 450008 China; Department of Hematology, Affiliated Cancer Hospital of Zhengzhou University and Henan Cancer Hospital, Zhengzhou, 450008 China

**Keywords:** Cytokine-induced killer cells, Immunotherapy, Pancreatic cancer, Overall survival

## Abstract

**Background:**

Advanced pancreatic cancer (PC) has very poor prognosis with present treatments, thus necessitating continued efforts to find improved therapeutic approaches. Both preclinical and preliminary clinical data indicate that cytokine-induced killer (CIK) cells are an effective tool against various types of solid tumors. Here, we conducted a study to determine whether CIK cell-based therapy (CBT) can improve the outcomes of advanced PC.

**Methods:**

Eighty-two patients with advanced PC, whose predicted survival time was longer than 3 months, were analyzed retrospectively. Of all the patients, 57 individuals were receiving chemotherapy, while the remaining 25 individuals were treated with CBT.

**Results:**

The overall survival analysis was based on 48 deaths in the 57 patients in the chemotherapy group (84.2 %) and 18 deaths in the 25 patients in the CBT group (72.0 %). In the CBT group, the median overall survival time was 13.5 months, as compared to 6.6 months in the chemotherapy group (hazard ratio for death, 0.39; 95 % confidence interval, 0.23 to 0.65; *p* < 0.001). The survival rate was 88.9 % in the CBT group versus 54.2 % in the chemotherapy group at 6 months, 61.1 % versus 12.5 % at 12 months, and 38.9 % versus 4.2 % at 18 months. The disease control rate was 68.0 % in the CBT group and 29.8 % in the chemotherapy group (*p* < 0.001).

**Conclusions:**

These results from this retrospective analysis appeared to imply that CBT might prolong survival in these high-risk PC patients. Prospective study is needed to corroborate this observation.

**Electronic supplementary material:**

The online version of this article (doi:10.1186/s13045-016-0237-6) contains supplementary material, which is available to authorized users.

## Background

Pancreatic cancer (PC) has the poorest prognosis among all gastrointestinal cancers, with 1-year survival rate of around 20 % and 5-year survival rate of 7 % for diagnosed patients [1–3]. Gemcitabine is a chemical agent used as standard chemotherapy treatment for advanced PC. However, patients treated with this agent alone have a median overall survival time (mOS) of no more than 8.3 months, and the results of most clinical trials show that the mOS of advanced PC patients is not significantly prolonged when gemcitabine is combined with other cytotoxic or targeted agents [3–5]. Patients who received FOLFIRINOX (oxaliplatin, irinotecan, fluorouracil, and leucovorin) or nab-paclitaxel combined with gemcitabine therapy show an improvement in mOS, with an increase of 4.3 or 1.8 months, respectively [2, 6]. However, these strategies are associated with a higher incidence of serious side effects, and the patients must therefore undergo rigorous testing prior to chemotherapy to have evaluated suitability for therapy, and further require close monitoring during the therapy. Thus, new therapeutic strategies for advanced PC treatment are urgently needed.

A promising approach treating PC is the use of immune checkpoint inhibitors. The targets of these treatments are the molecules that serve as checks in the regulation of immune responses. By blocking inhibitory molecules, these treatments will activate the immune system and as such reactivate preexisting anti-cancer responses [7–11]. Several checkpoint inhibitors are currently in development.

Another major avenue of immunotherapy for PC is adoptive T cell transfer. Cytokine-induced killer (CIK) cells are a heterogeneous subset of T lymphocytes expanded ex vivo that express CD3 and CD56 as well as natural killer group 2, member D molecules (NKG2D). Adoptive transfer of CIK cells has been shown to be effective for cancer treatment, with high safety, as indicated by the prolonged survival of patients with different types of tumors [12–17]. When used in combination with chemotherapy, CIK cells show enhanced efficacy in preventing disease recurrence and improving the prognosis of cancer patients [18–20]. Recently, researchers have been taking advantage of CIK cells as a second-line treatment to treat advanced PC, and the results obtained showed encouraging results, both in terms of single use and combined use with other treatments. For example, in a phase 2 clinical trial, patients with CIK cells in gemcitabine-refractory advanced PC displayed a mOS of 6.2 months [21]. Another clinical study showed that patients who received CIK cells in combination with S-1, an oral fluoropyrimidine derivative, in gemcitabine-refractory advanced PC displayed a mOS of 6.6 months, which is longer than mOS of patients receiving S-1 alone (6.1 months) [22]. However, data are lacking on the efficacy of CIK cells as a first-line treatment in patients with advanced PC.

In this study, we performed a retrospective study, where we compared the mOS of patients with advanced PC, who have been treated with CBT or chemotherapy as a first-line treatment.

## Results

### Characteristics of the patients

A total number of 82 patients were enrolled in this study. Of these, 57 patients received chemotherapy alone, and 25 patients received CBT. The last follow-up examination of the patients was performed on September 13, 2014. Although sample numbers were different in the two groups, the baseline demographic and clinical characteristics of patients were relatively well balanced (Table 1 and Additional files 1 and 2).

**Table 1:** Demographic and clinicopathological characteristics of the patients at baseline

Characteristics	Chemotherapy (*n* = 57)		CBT (*n* = 25)		*p* value
	Number	%	Number	%	
Sex					
Male	35	61.40	15	60.00	1.00
Female	22	38.60	10	40.00	
Age, years					
<65	38	66.67	14	56.00	0.46
≥65	19	33.33	11	44.00	
Diagnosis basis					
Pathologically	29	50.88	14	56.00	0.81
Clinically	28	49.12	11	44.00	
Extent of disease					
Locally advanced	9	15.79	2	8.00	0.49
Metastatic	48	84.21	23	92.00	
Measurable metastatic sites					
Liver^a^	30	52.63	13	52.00	1.00
Other	27	47.37	12	48.00	
No. of metastatic sites					
1	32	56.14	16	64.00	0.83
2	18	31.58	7	28.88	
≥3	7	12.28	2	8.00	
ECOG performance status score					
1	20	35.09	9	36.00	1.00
2	37	64.91	16	64.00	
Pancreatic tumor location					
Head	21	36.84	14	56.00	0.17
Body	5	8.78	4	16.00	
Tail	16	28.07	3	12.00	
Multicentric^b^	15	26.32	4	16.00	
Level of carbohydrate antigen 19-9 - u/ml					
Abnormal	47	82.46	20	80.00	0.23
Normal	6	10.53	5	20.00	
Unknown^c^	4	7.02	0	0.00	

^a^Patients with liver-only metastasis or liver-containing metastases were included

^b^When tumor infiltrated more than one region of pancreas, it was defined as multicentric. It included head-body, head-tail, body-tail, and head-body-tail tumors

^c^Four patients did not carry out this detection for economic condition

### Survival

**Fig. 1:**
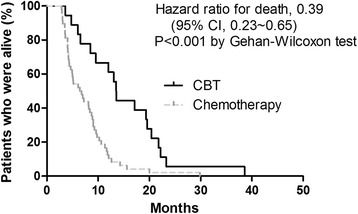
Overall survival time for CBT versus chemotherapy alone in patients with advanced PC. Overall survival was calculated in 18 patients with CBT compared with 48 patients with chemotherapy

The overall survival analysis was based on 48 deaths in the 57 patients in the chemotherapy group (84.2 %) and 18 deaths in the 25 patients in the CBT group (72.0 %). The mOS was 13.5 months in the CBT, as compared with 6.6 months in the chemotherapy group (hazard ratio for death, 0.39; 95 % CI, 0.23 to 0.65; *p* < 0.001) (Fig. 1). The overall survival rates at 6, 12, and 18 months were 88.9, 61.1, and 38.9 %, respectively, in the CBT group, as compared with the survival rate of 54.2, 12.5, and 4.2 % in the chemotherapy group.

### Response

**Fig. 2:**
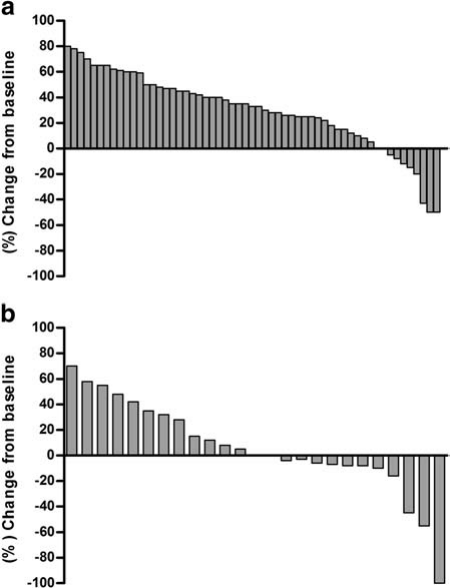
Waterfall chart demonstrating changes in tumor measurement with measurable tumor at baseline. Response to therapy was calculated in 57 patients with chemotherapy (**a**) compared with 25 patients with CBT (**b**)

**Fig. 3:**
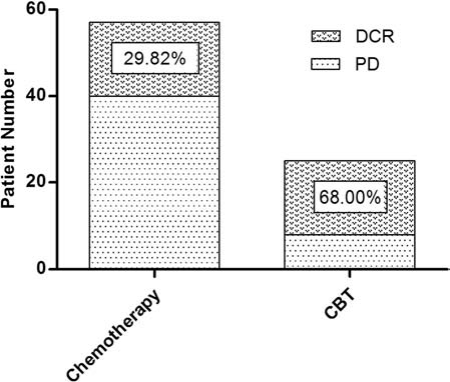
Comparison of the DCR rate in the CBT group and chemotherapy group

Of 57 patients in the chemotherapy group, no complete responses were seen, 3 patients exhibited a partial response (5.3 %), 14 patients displayed stable disease (24.6 %), and 40 patients showed progressive disease (70.2 %) (Fig. 2a). Of 25 patients in the CBT group, 1 complete response was seen (4.0 %), 2 patients exhibited a partial response (8.0 %), 14 patients displayed stable disease (56.0 %), and 8 patients showed progressive disease (32.0 %) (Fig. 2b). The DCR rate was 29.8 % (17/57) in the chemotherapy group and 68.0 % (17/25) in the CBT group. The DCR rate was significantly higher in the CBT group than in the chemotherapy group. (*p* < 0.001, Fig. 3).

### Adverse events

The major adverse events are listed in Table 2. Patients in the CBT group seemed to have higher incidences of fever and fatigue as compared with patients in the chemotherapy group. The difference was minimal, statistically not significant, and was considered to be attributed to the most common side effect of IL-2 administrated after CIK cell transfusion [14]. There were no significant differences in leucopenia, neutropenia, thrombocytopenia, nausea, diarrhea, vomiting, and infection between the two groups.

**Table 2:** Adverse events (AEs) among patients in chemotherapy and CBT groups

Adverse event	Chemotherapy (*n* = 57)		CBT (*n* = 25)		*p* value
	Number	%	Number	%	
Myelosuppression^a^	33	57.89	10	40.00	0.16
Digestive tract symptom^b^	18	31.58	5	20.00	0.42
Fever	2	3.51	3	12.00	0.16
Fatigue	2	3.51	2	8.00	0.58
Infection	2	3.51	0	0	1.00

^a^Myelosuppression includes thrombocytopenia, lymphopenia, and neutropenia

^b^Digestive tract symptom includes nausea, vomiting, and diarrhea

## Discussion

Overall survival time has traditionally been regarded as the most reliable endpoint in evaluating an experimental strategy for cancer treatment, and thus its improvement is an important criterion for regulatory approval of a new therapy. However, most large-scale clinical phase 3 studies have not shown significantly improved survival in advanced PC. Our data presented here suggest that in patients with advanced PC, the overall survival was significantly improved when the patients were treated with CBT, relative to patients who received chemotherapy alone. More importantly, CIK cell-associated toxicity was mild, with fever as the main side effect, at an incidence rate of approximately 15 % [14]. This suggests that CIK cell treatment has a higher efficacy and safety in advanced PC.

Gemcitabine and fluorouracil are two widely used chemotherapeutical agents in advanced PC. Previous studies have shown that gemcitabine suppresses the production of IgG antibody but does not affect the specific anti-tumor immunity [23, 24]. In another study, it was shown that the anti-tumor effect of gemcitabine does not result from direct cytotoxic effects on tumor cells but rather from an enhancement of T cell-mediated immune responses [25]. These data suggest that gemcitabine acts as an immunomodulator, making it a good candidate drug for combination with immunotherapy strategies. This notion is supported by two experimental studies showing that combination of a dendritic cell vaccine with gemcitabine improved the survival of tumor-bearing hosts in a murine pancreatic carcinoma model [26, 27]. Fluorouracil also exhibited immunomodulatory effects when combined with immunotherapy strategies [28]. Thus, the immunomodulatory effects of gemcitabine and fluorouracil play an important role for hosts to elicit robust anti-tumor immunity. On the basis of these data and our results, we propose that when combined with immunomodulatory chemotherapeutical agents, CIK cells exhibit even higher, more potent anti-tumor activity.

Previous in vitro experiments show that cultured CIK cells possess significant cytotoxic activity against tumor cells and most of the cytotoxicity is attributed to the higher proliferation of CD3^+^CD56^+^ cells [29, 30]. In vivo, CIK cells can migrate to the site of tumors through interacting with chemokine receptors expressed on the surface of them, where they release their cytotoxic potential and inhibit tumor growth [31–33]. The molecular mechanisms of tumor recognition and killing by CIK cells possibly involve the expression of lymphocyte function-associated antigens, NKG2D, DNAS accessory molecule-1, and NKp30 [29, 34]. Besides their direct cytotoxic effect, CIK cells secret interferon gamma that modulates the expression of adhesion molecules on tumor cells, and the altered expression pattern of adhesion molecule enhances apoptosis that is induced by cytotoxic effector cells [35]. In addition, recent studies report that CIK cells attack cancer stem cells in both animal models and human patients [13, 33]. Finally, previous work using a mouse model indicates that cancer immunotherapy using interferon alpha can significantly reduce chemotherapy-induced expression of multi-drug resistance proteins, as well as lower the activity of drug efflux out of the cancer cells subjected to this treatment, thus rescuing their chemosensitivity [36]. This result suggests that CIK cells increase the sensitivity of PC cells to gemcitabine and/or S-1, which is another possible mechanism for CIK cell anti-tumor action.

Recently, Arina and colleagues suggest that the presence of myeloid-derived suppressor cells (MDSC) limits adoptively transferred T cell infiltration and function, and manipulation of intratumoral myeloid cells may improve the outcome of otherwise unsuccessful adoptive transferred T cells [37]. Both gemcitabine and fluorouracil were known to be selectively cytotoxic on MDSC whereas no significant effect on T cells in animal models [38, 39], and MDSC decrease was observed when these chemotherapeutic agents were used in cancer patients [40, 41]. Thus, it is reasonable that CIK cells combined with these agents led to a better effect in advanced PC patients.

Our survival analysis has several major experimental limitations. First of all, this is a retrospective, non-randomized study, and only patients from medical wards of our hospital were included, which may not truly reflect patients from other hospitals. Moreover, survival differences between CBT and chemotherapy might be affected by differences during patient selection and differences in standard and supportive care treatment. A second limitation of our analysis is that we did not have detailed information on the post-chemotherapy treatment in the chemotherapy group, and this could contribute to the difference in mOS between the two groups. Thirdly, the use of chemotherapeutic agents in the two groups was not standardized. Despite this difference in chemical agents between the two groups, we believe that such difference had no significant influence on the difference in mOS, since the results of previous clinical trials clearly indicated that there was no significant increase in mOS when two or more drugs were combined. Thus, the observed therapeutic benefits in the combined group were considered to be a result of the transfusion with CIK cells.

## Conclusions

In summary, the fact that we observed prolonged survival for patients receiving CBT supports the notion that CIK cells can greatly improve the prognosis of advanced PC. Based on the data presented here, other estimates of clinical benefit such as objective response rate and progression-free survival and improved quality of life, CBT will hopefully become a standard of care for patients with advanced PC.

## Methods

### Patient selection

Patients in this retrospective analysis were admitted between 1 September 2010 and 4 May 2014 in Henan Cancer Hospital. The criteria for patients included in the study were the following: (1) PC was histologically or cytologically confirmed (pathologically diagnosed), or diagnosed by symptoms and complications, computer tomography or magnetic resonance imaging, combined with measurement of serum carbohydrate antigen 199 levels (clinically diagnosed). (2) The ECOG performance status was below 3. (3) The predicted survival time was longer than 3 months. (4) Age is larger than 18 years. (5) Uncontrolled infection was absent. Unresectability was assessed by an experienced surgeon either during laparotomy or by radiologic work-up (computed tomography scan and/or magnetic resonance imaging) showing portal and/or mesenteric and/or celiac vascular involvement. Any extrapancreatic disease including Vater’s ampulloma and adenocarcinoma of the biliary tract was an exclusion criterion. We listed all patients who met the above criteria and surveyed their records. All patients gave written informed consent, which had been approved by the institutional review board of Zhengzhou University. This study was conducted in accordance with the provisions of the Declaration of Helsinki and Good Clinical Practice guidelines.

### Study design

CIK cells were prepared according to the procedures described in a previous study [13]. Briefly, peripheral blood mononuclear cell were separated and cultured under sterile conditions with 1640 medium containing anti-CD3 monoclonal antibody, interferon gamma, interleukin-2, and RetroNectin (RN, Takara, Japan). After culturing of cells for 10 to 14 days, a target dose of about 5 × 10^9^ CIK cells with over 95 % viability was obtained and tested for biological contaminants. The modified method led to a significant higher proportion of CD3^+^CD56^+^ cellular subset [14]. Anti-tumor cytokines such as tumor necrosis factor alpha were higher and pro-tumor cytokines such as interleukin-4 and interleukin-5 were lower in the CIK cell cultures prepared with the modified method (data not shown). Cells were then prepared in 2 % albumin containing sodium chloride solution before transfusion into the patients. In the following 3 days, patients were administered with interleukin-2 (2 million IU per day) to promote CIK activity. Patients in this study received at least 2 cycles of CIK cell transfusion.

In the CBT group, 21 patients received CIK cell transfusion plus gemcitabine (1000 mg/m^2^, d1, 8) and/or S-1 (orally twice daily at a dose according to the body surface area (<1.25 m^2^, 80 mg/day; 1.25–1.5 m^2^, 100 mg/day; >1.5 m^2^, 120 mg/day) on days 1–14), one patient received CIK cell transfusion plus epotoside (60 mg/m^2^, d1-5), and three cases received only CIK cells. Transfusion of CIK cells was followed at the end of each cycle of chemotherapy. Patients received chemotherapy every 3-week period.

In the chemotherapy group, 54 patients received gemcitabine (1000 mg/m^2^, d1, 8) and/or S-1-based (orally twice daily at a dose according to the body surface area (<1.25 m^2^, 80 mg/day; 1.25–1.5 m^2^, 100 mg/day; >1.5 m^2^, 120 mg/day) on days 1–14) chemotherapy, one patient received epotoside (60 mg/m^2^, d1-5) and cisplatin (15 mg/m^2^, d2-5), one patient received nab-paclitaxel (110 mg/m^2^, d1, 8) and gemcitabine (1000 mg/m^2^, d1, 8), and one patient received an intraperitoneal perfusion of cisplatin (20 mg/m^2^, d1, 2). Patients received chemotherapy every 3-week period.

### Evaluation of short-term efficacy and toxicity

The tumor response was assessed according to the Response Evaluation Criteria in Solid Tumors (RECIST): complete response (CR), partial response (PR), stable disease (SD), and progressive disease (PD). We performed tumor assessments with the use of computed tomographic (CT) scanning at baseline and 2 months post treatment start. Efficacy was evaluated by disease control rate (DCR), consisting of CR, PR, and SD. Safety was assessed by documentation of adverse events. Hematologic and serum chemical measurement were performed before and after each cycle of treatment. Adverse events were graded with the use of the Common Terminology Criteria for Adverse Events of the National Cancer Institute, version 3.0.

### Statistical analysis

Fisher’s exact test or chi-square test were used to assess the association of the treatment group with demographic and clinicopathological characteristics as well as the response to therapy. Overall survival was estimated using the Kaplan-Meier method, and the log-rank test was used to compare the difference between the two groups. The hazard ratio between the two groups was estimated by proportional hazards regression using a 95 % Wald confidence interval (95 % CI). Data analysis was done with Graphpad Prism 5, with all *p* values applied as two-sided.

## Additional files

Additional file 1:
**Demographic and clinical characteristics of individual patient in chemotherapy group.** (DOC 85 kb) Additional file 2:
**Demographic and clinical characteristics of individual patient in CBT group.** (DOC 43 kb)

## Abbreviations

CBT: cytokine-induced killer cell-based therapy
CIK: cytokine-induced killer
DCR: disease control rate
MDSC: myeloid-derived suppressor cells
mOS: median overall survival time
PC: pancreatic cancer
