# Supplementary material for: Autologous cytokine-induced killer cell transfusion increases overall survival in advanced pancreatic cancer
Source: J Hematol Oncol. 2016 Feb 3;9:6. doi: 10.1186/s13045-016-0237-6 (PMC4740990; doi:10.1186/s13045-016-0237-6)
Supplement: Additional file 1: — Demographic and clinical characteristics of individual patient in chemotherapy group. (DOC 85 kb) [file 13045_2016_237_MOESM1_ESM.doc]

| Patient No | Age/Sex | Diagnosed date | Metastases | Therapy | Outcome |
| --- | --- | --- | --- | --- | --- |
| 1 | 78/F | 2011.12.12 | Lung liver mediastinum | Chemotherapy(S-1 Gem) | PD died on month 20.03 |
| 2 | 63/F | 2012.02.11 | Liver | Chemotherapy(Gem) | PD died on month 11.33 |
| 3 | 63/M | 2012.03.07 | / | Chemotherapy(Gem+5-FU) | PD died on month 8.10 |
| 4 | 39/M | 2012.08.11 | / | Chemotherapy(Gem) | PD died on month 15.63 |
| 5 | 56/M | 2012.07.25 | Liver LN | Chemotherapy(Gem+OXA CPT-11+S-1) | PD died on month 14.30 |
| 6 | 70/M | 2012.08.22 | Liver | Chemotherapy(S-1 DDP) | PD died on month 9.07 |
| 7 | 71/F | 2012.08.21 | Enterocoelia liver | Chemotherapy(DDP) | PD died on month 3.10 |
| 8 | 76/F | 2012.05.10 | / | Chemotherapy(S-1) | SD died on month 9.30 |
| 9 | 58/M | 2013.01.07 | / | Chemotherapy(Gem+5-FU) | SD died on month 9.03 |
| 10 | 67/F | 2013.01.29 | Liver | Chemotherapy(Gem+DDP) | PD died on month 7.00 |
| 11 | 54/M | 2012.12.05 | Liver | Chemotherapy(Gem+OXA) | PD died on month 5.00 |
| 12 | 60/F | 2013.01.08 | Liver | Chemotherapy(Gem+S-1) | PD died on month 3.50 |
| 13 | 66/M | 2013.01.05 | Liver | Chemotherapy(Gem+OXA) | PD died on month 5.13 |
| 14 | 55/M | 2013.02.09 | Enterocoelia liver | Chemotherapy(Gem+DDP) | PD died on month 4.20 |
| 15 | 62/F | 2013.03.22 | Liver Adrenal | Chemotherapy(Gem+OXA Gem+CAP) | SD died on month 6.47 |
| 16 | 50/M | 2013.02.21 | / | Chemotherapy(Gem+OXA) | SD alive on 18.97months |
| 17 | 45/F | 2013.03.12 | Liver | Chemotherapy(Gem+DDP Gem+5-FU) | PD died on month 10.20 |
| 18 | 65/M | 2012.04.20 | Liver | Chemotherapy(Gem) | PD died on month 3.60 |
| 19 | 66/M | 2012.01.19 | Abdominal wall | Chemotherapy(Gem+OXA) | SD died on month 6.13 |
| 20 | 75/F | 2011.11.12 | Enterocoelia LN | Chemotherapy(Gem+DDP) | PD died on month 3.57 |
| 21 | 55/M | 2011.11.08 | / | Chemotherapy(Gem 5-FU) | SD alive on 34.67months |
| 22 | 75/F | 2011.08.01 | Liver LN | Chemotherapy(Gem+DDP) | PD died on month 6.80 |
| 23 | 45/M | 2011.05.17 | Enterocoelia LN | Chemotherapy(Gem) | PD died on month 4.33 |
| 24 | 70/M | 2011.07.16 | Liver | Chemotherapy(Gem) | PD died on month 11.83 |
| 25 | 62/M | 2011.09.28 | Adrenal Spleen LN | Chemotherapy(Gem) | PD died on month 9.60 |
| 26 | 61/M | 2011.07.26 | Liver | Chemotherapy(Gem) | PD died on month 4.10 |
| 27 | 58/F | 2011.12.08 | LN | Chemotherapy(Gem Gem+OXA) | PD died on month 4.07 |
| 28 | 38/M | 2011.09.09 | Liver Spleen | Chemotherapy(Gem+OXA) | SD died on month 8.73 |
| 29 | 50/F | 2011.10.06 | Liver | Chemotherapy(Gem+DDP Gem) | PD died on month 12.60 |
| 30 | 56/M | 2011.07.15 | Liver LN | Chemotherapy(Gem+OXA) | PD died on month 8.90 |
| 31 | 61/F | 2011.06.08 | Enterocoelia | Chemotherapy(Gem) | PD died on month 3.57 |
| 32 | 74/F | 2011.05.10 | Liver LN | Chemotherapy(Gem+DDP) | SD died on month 8.37 |
| 33 | 42/M | 2011.05.04 | Liver Bone | Chemotherapy(Gem+OXA) | SD died on month 3.07 |
| 34 | 55/F | 2011.06.07 | Liver | Chemotherapy(Gem) | PD died on month 29.87 |
| 35 | 42/F | 2011.01,25 | Liver Lung Brain LN | Chemotherapy(Gem+DDP) | PD died on month 4.03 |
| 36 | 82/M | 2011.03.05 | Liver Lung LN | Chemotherapy(Gem+CAP) | PD died on month 4.67 |
| 37 | 59/M | 2011.03.29 | Liver Adrenal LN | Chemotherapy(Gem+DDP Gem) | SD died on month 12.07 |
| 38 | 77/F | 2011.03.01 | / | Chemotherapy(Gem S-1) | PD died on month 11.67 |
| 39 | 69/F | 2010.11.18 | Liver | Chemotherapy(Gem+DDP) | PD died on month 4.20 |
| 40 | 55/M | 2010.11.12 | Liver Adrenal | Chemotherapy(Gem+OXA) | PD died on month 4.07 |
| 41 | 57/F | 2013.04.01 | Liver | Chemotherapy(Gem+OXA) | PD died on month 9.80 |
| 42 | 59/M | 2013.04.01 | Lung Enterocoelia | Chemotherapy(Gem+ S-1) | PD died on month 7.23 |
| 43 | 41/M | 2013.07.01 | Liver | Chemotherapy(Gem+DDP YH-16+DDP+S-1) | PR alive on 14.57months |
| 44 | 62/M | 2013.03.11 | Spleen Enterocoelia | Chemotherapy(Gem+S-1) | PD died on month 10.60 |
| 45 | 66/F | 2013.09.12 | / | Chemotherapy(Gem+OXA) | SD alive on 11.87months |
| 46 | 55/M | 2013.07.28 | Liver Bone | Chemotherapy(Gem+S-1) | PD died on month 4.93 |
| 47 | 67/M | 2013.07.28 | Liver LN | Chemotherapy(Gem) | SD died on month 3.07 |
| 48 | 44/M | 2013.08.22 | Liver | Chemotherapy(Gem+DDP) | SD alive on 12.90months |
| 49 | 60/F | 2013.09.26 | / | Chemotherapy(Gem+OXA DOC+S-1) | PD died on month 4.40 |
| 50 | 56/M | 2013.10.21 | Liver | Chemotherapy(VP-16+DDP VP-16) | PD alive on 10.90months |
| 51 | 56/M | 2013.11.15 | Liver | Chemotherapy(Gem+OXA S-1) | PD died on month 8.17 |
| 52 | 74/M | 2013.12.17 | Liver Lung | Chemotherapy(Gem+DDP) | PR died on month 2.90 |
| 53 | 51/M | 2014.01.10 | Liver | Chemotherapy(Gem+PTX) | PR alive on 8.20months |
| 54 | 71/M | 2013.12.25 | Liver LN | Chemotherapy(Gem+Nimotuzumab) | SD alive on 8.73months |
| 55 | 63/F | 2014.01.21 | Pelvic cavity | Chemotherapy(Gem+OXA) | PD died on month 2.90 |
| 56 | 49/M | 2014.1.12 | Liver Adrenal LN | Chemotherapy(FOLFOX4 Gem+DDP Gem+S-1) | PD alive on 8.13 months |
| 57 | 53/M | 2010.09.01 | Liver | Chemotherapy(Gem) | PD died on month 5.17 |
|  |  |  |  |  |  |
|  |  |  |  |  |  |

LN lymph nodes
