# Supplementary material for: Autologous cytokine-induced killer cell transfusion increases overall survival in advanced pancreatic cancer
Source: J Hematol Oncol. 2016 Feb 3;9:6. doi: 10.1186/s13045-016-0237-6 (PMC4740990; doi:10.1186/s13045-016-0237-6)
Supplement: Additional file 2: — Demographic and clinical characteristics of individual patient in CBT group. (DOC 43 kb) [file 13045_2016_237_MOESM2_ESM.doc]

| Patient No | | Age/Sex | Diagnosed date | Metastases | Therapy | Outcome |
| --- | --- | --- | --- | --- | --- | --- |
| 1 | 61/F | | 2010.09.24 | Liver | Chemotherapy(S-1)+CIK | PD died on month 3.63 |
| 2 | 58/M | | 2012.05.09 | Liver | Chemotherapy(S-1 Gem+OXA)+CIK | PD died on month 4.80 |
| 3 | 76/F | | 2011.0917 | Liver | CIK | SD died on month 13.50 |
| 4 | 49/M | | 2012.06.24 | Liver | Chemotherapy(S-1)+CIK | SD died on month 8.50 |
| 5 | 49/F | | 2011.11.08 | Liver LN | Chemotherapy(S-1)+CIK | SD died on month 13.03 |
| 6 | 70/M | | 2010.09.01 | Lung | Chemotherapy(Gem)+CIK | SD died on month 12.17 |
| 7 | 71/M | | 2011.01.17 | Liver Adrenal | Chemotherapy(S-1)+CIK | SD died on month 22.20 |
| 8 | 58/M | | 2011.09.07 | Enterocoelia | Chemotherapy(Gem+OXA)+CIK | SD died on month 13.53 |
| 9 | 52/M | | 2012.04.21 | Stomach Duodenum | CIK | SD alive on29.17 months |
| 10 | 77/F | | 2012.05.28 | / | CIK | CR died on month19.37 |
| 11 | 79/F | | 2010.11.15 | Liver Bone | CIK | SD died on month 23.33 |
| 12 | 72/M | | 2011.05.21 | Liver | Chemotherapy(S-1)+CIK | PR died on month19.57 |
| 13 | 74/F | | 2011.09.22 | Lung | Chemotherapy(S-1)+CIK | PR died on month20.43 |
| 14 | 25/F | | 2014.05.04 | Liver LN | Chemotherapy(S-1 Gem)+CIK | SD alive on 7.50 months |
| 15 | 75/M | | 2014.03.24 | Enterocoelia | Chemotherapy(S-1 Gem)+CIK | PD alive on 5.77months |
| 16 | 34/F | | 2014.03.25 | / | Chemotherapy(S-1 Gem)+CIK | SD alive on 5.70months |
| 17 | 66/M | | 2013.03.26 | Lung | Chemotherapy(Gem)+CIK | PD died on month 9.53 |
| 18 | 49/M | | 2013.10.09 | Liver | Chemotherapy(VP-16+DDP)+CIK | SD alive on 14.40months |
| 19 | 46/M | | 2012.03 | Enterocoelia Liver LN | Chemotherapy(Gem+DDP)+CIK | PD died on month 21.80 |
| 20 | 64/F | | 2014.03.22 | Liver | Chemotherapy(S-1)+CIK | PD died on month 6.57 |
| 21 | 72/F | | 2014.02.21 | Enterocoelia Liver | Chemotherapy(S-1+Gem)+CIK | PD died on month 6.03 |
| 22 | 58/M | | 2012.01.24 | Enterocoelia Liver | Chemotherapy(Gem)+CIK | SD died on month 17.10 |
| 23 | 51/M | | 2010.12.25 | Liver | Chemotherapy(Gem+OXA)+CIK | SD died on month 38.60 |
| 24 | 54/M | | 2013.10.23 | Lung Brain Enterocoelia | Chemotherapy(S-1+Gem)+CIK | SD alive on 10.83 months |
| 25 | 67/M | | 2014.03 | lung | Chemotherapy(S-1+Gem)+CIK | PD alive on 6.53 months |

LN lymph nodes
